# Phenotyping Adopters of Mobile Applications Among Patients With COPD: A Cross-Sectional Study

**DOI:** 10.3389/fresc.2021.729237

**Published:** 2021-11-04

**Authors:** Sofia Flora, Nádia Hipólito, Dina Brooks, Alda Marques, Nuno Morais, Cândida G. Silva, Fernando Silva, José Ribeiro, Rúben Caceiro, Bruno P. Carreira, Chris Burtin, Sara Pimenta, Joana Cruz, Ana Oliveira

**Affiliations:** ^1^Center for Innovative Care and Health Technology, Polytechnic of Leiria, Leiria, Portugal; ^2^School of Rehabilitation Science, McMaster University, Hamilton, ON, Canada; ^3^West Park Healthcare Centre, Toronto, ON, Canada; ^4^Respiratory Research and Rehabilitation Laboratory, School of Health Sciences (ESSUA), University of Aveiro, Aveiro, Portugal; ^5^Institute of Biomedicine, University of Aveiro, Aveiro, Portugal; ^6^School of Health Sciences, Polytechnic of Leiria, Leiria, Portugal; ^7^Centre for Rapid and Sustainable Product Development, Polytechnic Institute of Leiria, Leiria, Portugal; ^8^Department of Chemistry, Coimbra Chemistry Centre, University of Coimbra, Coimbra, Portugal; ^9^School of Technology and Management, Computer Science and Communications Research Centre, Polytechnic Institute of Leiria, Leiria, Portugal; ^10^Unidade de Saúde Familiar Pedro e Inês, ACeS Oeste Norte, Alcobaça, Portugal; ^11^Faculty of Rehabilitation Sciences, REVAL—Rehabilitation Research Center, Hasselt University, Diepenbeek, Belgium; ^12^BIOMED—Biomedical Research Institute, Hasselt University, Diepenbeek, Belgium

**Keywords:** COPD, mHealth, mobile apps, physical activity, smartphones

## Abstract

Effectiveness of technology-based interventions to improve physical activity (PA) in people with COPD is controversial. Mixed results may be due to participants' characteristics influencing their use of and engagement with mobile health apps. This study compared demographic, clinical, physical and PA characteristics of patients with COPD using and not using mobile apps in daily life. Patients with COPD who used smartphones were asked about their sociodemographic and clinic characteristics, PA habits and use of mobile apps (general and PA-related). Participants performed a six-minute walk test (6MWT), gait speed test and wore an accelerometer for 7 days. Data were compared between participants using (App Users) and not using (Non-App Users) mobile apps. A sub-analysis was conducted comparing characteristics of PA–App Users and Non-Users. 59 participants were enrolled (73% Male; 66.3 ± 8.3 yrs; FEV_1_ 48.7 ± 18.4% predicted): 59% were App Users and 25% were PA-App Users. Significant differences between App Users and Non-App Users were found for age (64.2 ± 8.9 vs. 69.2 ± 6.3yrs), 6MWT (462.9 ± 91.7 vs. 414.9 ± 82.3 m), Gait Speed (Median 1.5 [Q1–Q3: 1.4–1.8] vs. 2.0 [1.0–1.5]m/s), Time in Vigorous PA (0.6 [0.2–2.8] vs. 0.14 [0.1–0.7]min) and Self-Reported PA (4.0 [1.0–4.0] vs. 1.0 [0.0–4.0] Points). Differences between PA–App Users and Non-Users were found in time in sedentary behavior (764.1 [641.8–819.8] vs. 672.2 [581.2–749.4] min) and self-reported PA (4.0 [2.0–6.0] vs. 2.0 [0.0–4.0] points). People with COPD using mobile apps were younger and had higher physical capacity than their peers not using mobile apps. PA-App Users spent more time in sedentary behaviors than Non-Users although self-reporting more time in PA.

## Introduction

People with COPD present persistent airflow limitation, respiratory symptoms, such as dyspnea and fatigue, and exercise intolerance (1) which greatly impact their daily life (2). These symptoms make physical activity (PA) an unpleasant experience, which many patients try to avoid, leading to inactive lifestyles (3). Indeed, people with COPD commonly present lower levels of PA (1) than age- and sex-matched healthy peers and patients with other non-communicable diseases (4).

PA is defined as “any bodily movement produced by skeletal muscles that results in energy expenditure,” including exercise (a planned and structured type of PA) and everyday life activities (5). Low PA levels are the 4th leading risk factor for death worldwide (6) and, in people with COPD, they are highly associated with increased risk for hospitalizations, mortality and reduced health-related quality of life (1, 4). Thus, improving patients' PA levels is a priority for patients themselves, governments, policymakers and clinicians worldwide (1, 7).

Despite PA promotion being a part of COPD management guidelines (1, 4, 7), it remains a challenge for clinicians and researchers to operationalize effective and sustainable ways to increase PA levels and maintain them in the long term (1, 8). The use of technology-based interventions has gained popularity over the years to improve PA levels in COPD and in other chronic diseases (1, 9, 10), including mobile health (mHealth) apps (8, 9, 11, 12). However, studies conducted in COPD yielded mixed results for improvements in PA, exercise and health-related quality of life outcomes (8, 9, 12), which may be related to participants' characteristics that influence their adherence and engagement with mHealth apps.

It has been shown that users of mHealth apps are mostly younger people with higher income, higher education and self-reports of excellent health and PA levels (13). Comprehensive knowledge of the potential end-users' characteristics is key to personalize the design and marketing of mobile apps aiming at maximizing its use. Particularly in people with COPD, such knowledge may potentiate long-term adherence to the PA-enhancing intervention and consequently its effect on PA outcomes. However, the characteristics of people with COPD that utilize/do not utilize mobile apps in daily life have not been explored.

The aim of this study was to compare demographic, clinical, physical and PA characteristics of patients with COPD who report using mobile apps in daily life with those who do not use them.

## Methods

### Study Design

This was a prospective cross-sectional study conducted as part of a larger study (OnTRACK, ref. POCI-01-0145-FEDER-028446; PTDC/SAU-SER/28446/2017). Ethical approval was obtained prior to data collection from the Ethics Committees of Polytechnic of Leiria, the Hospital Centres of Leiria and Baixo Vouga, the District Hospital of Figueira da Foz, the Northern Lisbon University Hospital Centre, and the Regional Health Administration of Central Portugal. This paper follows the guidelines for STrengthening the Reporting of OBservational studies in Epidemiology (STROBE) (14).

### Participants

Potential participants were recruited from Hospitals and a primary care center (USF Santiago, Leiria) collaborating in the study. Individuals were included if they were: smartphone users; 18 years old or more; diagnosed with COPD according to the GOLD criteria (1); clinically stable in the previous month (i.e., no hospital admissions or acute exacerbations); fluent in Portuguese and able to provide informed consent. Exclusion criteria were the presence of severe neurologic, musculoskeletal, or psychiatric disorders, unstable cardiovascular disease, or other health condition/impairment that could preclude patients from understanding the study and/or participating in data collection. Eligible individuals were identified by the clinicians working in the recruitment institutions who informed them about the study. Those who expressed interest in participating were contacted via phone call by a member of the research team who provided additional information. Informed consent was obtained on the day of the assessment prior to any data collection.

### Data Collection

Data were collected at the Centre for Innovative Care and Health Technology (ciTechCare) of the Polytechnic of Leiria, at the Respiratory Research and Rehabilitation Laboratory—School of Health Sciences, University of Aveiro (Lab3R-ESSUA), or at the health units, according to participants' and services' availability.

Participants completed a structured questionnaire that included sociodemographic characteristics (age, sex and education level), general clinical information [height and weight to calculate body mass index (BMI), percentage of the predicted forced expiratory volume in one second (FEV_1_% predicted), comorbidities and exacerbation history], habits of using mobile apps and interest in using a COPD specific mobile app for PA promotion in the future. Participants were divided according to their answers into: (1) no use of mobile apps, i.e., the smartphone was only used for messaging (*via* SMS) or phone calls (using the standard call interface provided by the phone) (Non-App Users); and (2) use of any mobile app, such as social (i.e., Facebook, Instagram, Strava) and communication mobile apps (i.e., WhatsApp, Messenger, and Skype) independently of utilization frequency (App Users). App Users were further asked if they used mobile apps specifically for PA promotion (PA App Users).

Comorbidities were recorded by patient report, scored according to the Charlson Comorbidity Index and interpreted as mild (CCI scores of 1-2), moderate (CCI scores of 3-4) or severe (CCI scores ≥5) (15). Activities limitations related to dyspnea and the impact of COPD on health status were assessed with the modified Medical Research Council dyspnea scale (mMRC) (16) and the COPD Assessment Test (CAT) (17), respectively. The ABCD assessment tool was calculated using data from the exacerbation history and the mMRC (1). This tool allows to allocate participants into four categories: A—low exacerbations and low symptoms; B—low exacerbations and high symptoms; C—high exacerbations and low symptoms; and D—high exacerbations and high symptoms. Fatigue severity was assessed with the Portuguese version of the Checklist of Individual Strength (CIS20-P) (18).

Lung function was measured with a portable spirometer (MicroLoop, CareFusion, Kent, UK) according to the European guidelines (19) and the level of airflow obstruction limitation was established using the GOLD grades 1−4 (1). Exercise tolerance and gait speed were assessed with the 6-minute walking test (6MWT) (1, 20) and a 4.57-m (15 ft) gait speed test (21), respectively.

The Brief Physical Activity Assessment Tool (BPAAT) and accelerometry (22, 23) were used to assess participants' self-reported and objective levels of PA, respectively. The BPAAT is a simple, quick, and validated (22) questionnaire to use in clinical practice. It allows classifying patients in insufficiently (score < 4) or sufficiently active (score ≥ 4). The triaxial accelerometer ActiGraph GT3X+ (Pensacola, FL) was chosen as it has already been validated in COPD (24, 25). At the time of data collection, participants received the device and verbal and written instructions for its use. Instructions included using the device at the waist (on the dominant side) during waking hours, except for bathing or swimming, for 7 days. The Actigraph GT3X+ collects and stores PA data which can be downloaded and converted into time-stamped PA and step counts using specific software (ActiLife 6, v6.13.3, Pensacola, FL). Data were recorded at 1-min epoch intervals and then analyzed using the algorithms of Freedson et al. (26). Data on daily time (in min) spent in sedentary behavior [ <100 counts-per-minute (CPM)], light-intensity PA (100−1,951 CPM), moderate PA (1,952−5,724 CPM), vigorous PA (≥5,725 CPM), and a combination of moderate and vigorous PA (MVPA) (26) were extracted. The number of steps per day was also collected. Data were only considered valid if a minimum of 8 h/day for 4 weekdays of readings could be extracted from the device (27).

### Data Analysis

Descriptive statistics were used to characterize the sample. Normality of data distribution was assessed using Kolmogorov-Smirnov test. Chi-square, Mann-Whitney *U*- and *t*-tests [according to the (non-)normality of data distribution] were used to compare sociodemographic (age, sex and education level), health-related (FEV_1_% predicted, CAT, mMRC, 6MWT, gait speed test) and PA [step count, time spent (min/day) in sedentary behavior and each intensity of PA] characteristics between App Users and Non-App Users. A subgroup analysis was conducted to compare characteristics of PA Apps Users and Non-PA Apps Users. Effect sizes (ES) for the differences between groups were calculated as absolute values using the Hedge's g for normally distributed continuous data, *r* for non-normally distributed and ordinal data and the Cramer's V coefficient (Φ_c_) for categorical data (28, 29). Interpretation of effect sizes was small (<0.5), moderate (0.5−0.8) and large (≥0.8) for the Hedges' g ES (29) and small (<0.1), moderate (0.1−0.5) and large (≥0.5) for the *r* and Φ_c_ coefficients (28).

All data were analyzed using the Statistical Package for Social Sciences (SPSS)® software version 24 (IBM Corp., Armonk, USA) and statistical significance was considered at *p* < 0.05.

## Results

### Participants

**Figure 1:**
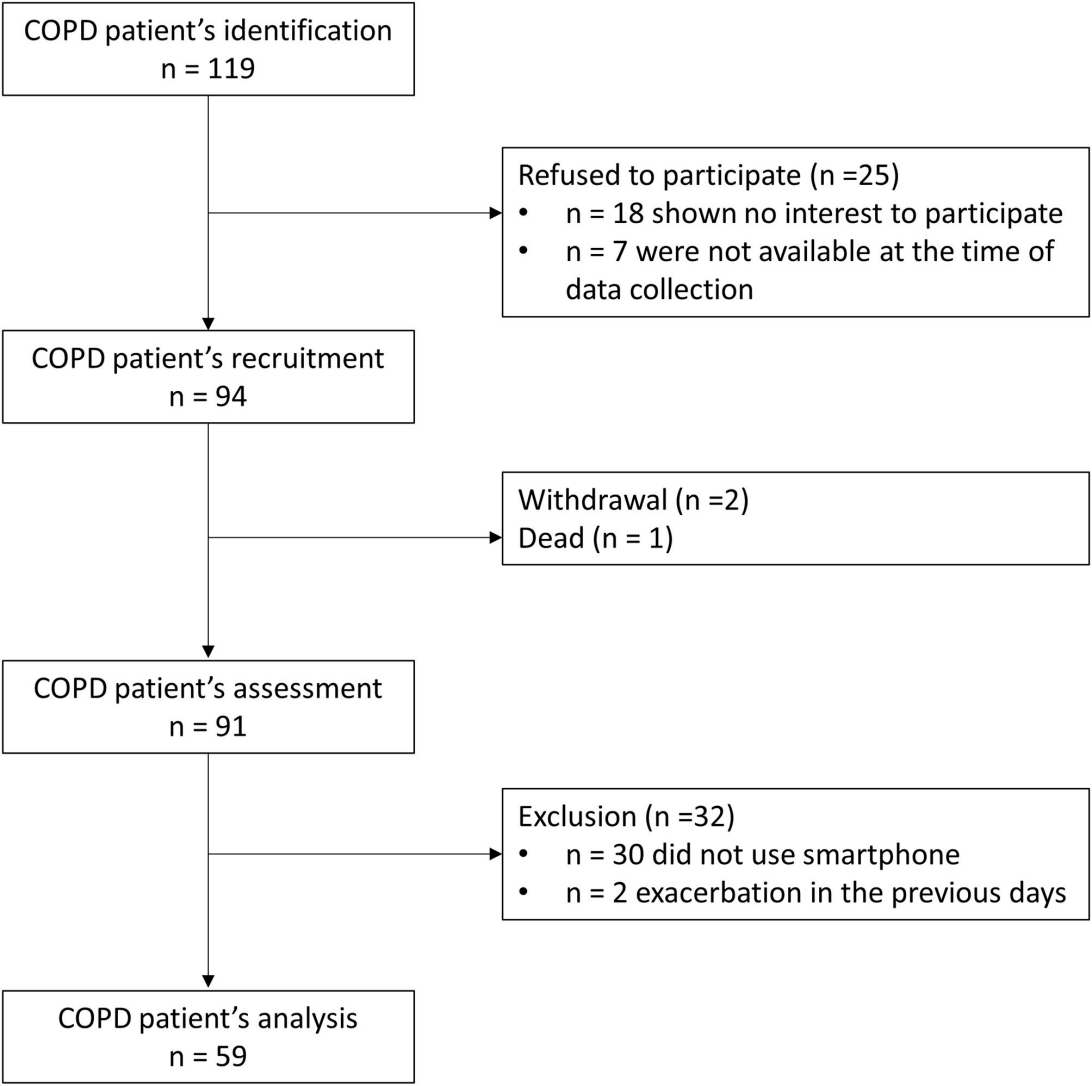
Flow chart of the participants' enrolment.

A total of 89 patients with COPD were invited to participate. From these, 25 refused to participate (18 had no interest in participating after receiving more information about the study and 7 were not available at the time of data collection), two withdrew from participating and 1 died from the moment of recruitment to consenting. Two participants were excluded due to having had exacerbations in the month prior to data collection. The final sample was composed of 59 participants (Figure 1). From these, 59% used mobile apps (App Users, *n* = 35) and a subgroup of 15 participants (25% of the total sample) used PA promotion apps, specifically. When questioned about the interest in using a mobile app to PA promotion specific to COPD in the future, 73% (*n* = 43) of participants answered positively.

Sample characteristics are in Table 1. Participants had a mean age of 66.3 ± 8.3 years, were mainly men (*n* = 43, 73%), have completed the primary education level (*n* = 39, 66%) and reported having moderate comorbidities (*n* = 36, 61%). Most participants presented moderate (*n* = 24, 41%, GOLD 2) or severe (*n* = 24, 41%, GOLD 3) airway obstruction and were grade A on the ABCD assessment tool (*n* = 28, 51%). Considering PA habits, 39% (*n* = 23) performed moderate PA for at least 30 min/day and 6.8% (*n* = 4) performed vigorous PA for at least 10 min/day. All participants wore the accelerometer for 8 h/day during 7 days.

**Table 1:** Participants' sociodemographic and clinical characteristics.

**Characteristics**	**Total** **(*n* = 59)**	**App Users** **(*n* = 35)**	**Non-App Users** **(*n* = 24)**	***P*-value**	**Effect** **size**
Age (years)	66.3 ± 8.3	64.2 ± 8.9	69.2 ± 6.3	0.023^*^	0.629
FEV_1_% pred	48.7 ± 18.4	50.8 ± 17.8	45.7 ± 19.3	0.303	0.277
BMI (kg/m^2^)	26.1 ± 4.8	26.5 ± 4.5	25.4 ± 5.4	0.412	0.225
Sex, *n* (%)				0.762	0.040
Female	16 (27)	10 (29)	6 (25)		
Male	43 (73)	25 (71)	18 (75)		
Education level, *n* (%)				0.050	0.969
Primary	39 (66.1)	22 (62.8)	17 (70.8)		
Secondary	11 (18.6)	8 (22.9)	3 (12.5)		
Undergraduate	2 (3.4)	2 (5.7)	0 (0)		
Graduate	7 (11.9)	3 (8.6)	4 (16.7)		
GOLD classification, *n* (%)				0.818	0.225
GOLD A	28 (50.9)	17 (53.1)	11 (47.8)		
GOLD B	8 (14.5)	5 (15.6)	3 (13)		
GOLD C	8 (14.5)	5 (15.6)	3 (13)		
GOLD D	11 (20.0)	5 (15.6)	6 (26.1)		
GOLD classification, *n* (%)				0.928	0.153
GOLD 1	2 (3.4)	1 (2.9)	1 (4.2)		
GOLD 2	24 (41.4)	15 (44.1)	9 (37.5)		
GOLD 3	24 (41.4)	14 (41.2)	10 (41.7)		
GOLD 4	8 (13.8)	4 (11.8)	4 (16.7)		
CCI, *n* (%)				0.508	0.216
Mild	10 (16.9)	7 (20.0)	4 (16.7)		
Moderate	36 (61.0)	19 (54.3)	17 (70.8)		
Severe	13 (22.0)	9 (25.7)	3 (12.5)		
CAT total score	13.1 ± 7.9	11.9 ± 6.8	14.8 ± 9.1	0.180	0.371
CIS20-P total	63.5 ± 23.9	59.3 ± 18.9	70.0 ± 27.9	0.142	0.466
mMRC (median [Q1; Q3])	1 [1; 2]	1 [1; 2]	1 [1; 2]	0.213	0.301

*Results are presented as mean ± standard deviation, unless otherwise stated*.

* *p < 0.05*.

*BMI, body mass index; CCI, Charlson Comorbidity Index; CAT, COPD Assessment Test; CIS20-P, Checklist of Individual Strength; FEV_1_, forced expiratory volume in the first second; GOLD, Global Initiative for Obstructive Lung Disease; mMRC, Modified Medical Research Council. Sample size per variable can be found in Supplementary Material 1*.

### Characteristics of “App Users” and “Non-App Users”

#### Sociodemographic and Clinical Characteristics

Characteristics of App Users and Non-App Users are presented in Table 1. Participants using apps were younger (64.2 ± 8.9 vs. 69.2 ± 6.3; *p* = 0.023; ES = 0.629) than those not using apps. A large effect size was found for differences between groups' education levels (Φ_c_ = 0.969). No differences were observed for the remaining sociodemographic and clinical characteristics (*p* > 0.05).

#### Physical Activity and Physical Capacity

Accelerometer-based data showed that App Users spent more time in vigorous activities (median [Q1; Q3]: 0.6 [0.2; 2.8] min/day vs. 0.14 [0.1; 0.7] min/day; *p* = 0.026; ES = 0.290) and self-reported higher levels of MVPA in the BPAAT (median [Q1; Q3]: 4.0 [1.0; 4.0] vs. 1.0 [0.0; 4.0] points; *p* = 0.002; ES = 0.397) than Non-App Users. No statistically significant differences were observed for the remaining PA variables (*p* > 0.05). App Users walked a greater distance in the 6MWT (462.9 ± 91.7 m vs. 414.9 ± 82.3 m; *p* = 0.047; ES = 0.545) and faster in the gait speed test (median [Q1; Q3]: 1.5 [1.4; 1.8] vs. 2.0 [1.0; 1.5] m/s; *p* = 0.003; ES = 0.334) than Non-App Users. A detailed description of PA and physical capacity characteristics for both groups can be found in Table 2.

**Table 2:** Physical activity and physical capacity of App Users and Non-App Users.

**Variable**	**App Users** **(*n* = 35)**	**Non-App Users** **(*n* = 24)**	***P*-value**	**Effect size**
**Physical activity**				
BPAAT	4.0 [1.0; 4.0]	1.0 [0.0; 4.0]	0.002^*^	0.397
Accelerometry (min/day)				
Sedentary behavior	673.6 [605.1; 776.6]	672.5 [614.3; 745.8]	0.974	0.004
Light PA	97.5 [83.0; 167.7]	117.2 [68.4; 141.5]	0.883	0.019
Moderate PA	28.7 [12.1; 52.5]	12.1 [6.5; 35.4]	0.145	0.190
Vigorous PA	0.6 [0.2; 2.8]	0.14 [0.1; 0.7]	0.026^*^	0.290
MVPA	30.7 [12.2; 55.4]	12.4 [6.7; 36.2]	0.095	0.218
Steps per day	5352 [3350; 8167]	3612 [2415; 6343]	0.154	0.186
**Physical capacity**				
6MWD (m)	462.9 ± 91.7	414.9 ± 82.3	0.047^*^	0.545
Gait speed (m/s)	1.5 [1.4; 1.8]	2.0 [1.0; 1.5]	0.010^*^	0.334

*Results are presented as mean ± standard deviation for variables that follow a normal distribution, and as median [P25; P75] for variables not following a normal distribution. Accelerometry-based data are a mean of 7 days. 6MWD, 6-min walking distance; BPAAT, Brief Physical Activity Assessment Tool; MVPA, moderate-to-vigorous physical activity; PA, physical activity. Sample size per variable is in Supplementary Material 1*.

* *p < 0.05*.

### Characteristics of “PA App Users” and “Non-PA App Users”

No significant differences were found between groups for the sociodemographic and clinical variables (*p* > 0.05; Supplementary Material 2). PA-App Users (*n* = 15) reported higher PA levels in the BPAAT (median [Q1; Q3]: 4.0 [2.0; 6.0] vs. 2.0 [0.0; 4.0], *p* = 0.016; ES = 0.313) than Non-PA App Users (*n* = 44). However, accelerometer-based data showed no between-group differences for objectively measured PA (*p* > 0.05). PA-App Users spent significantly more time in sedentary behavior than Non-PA App Users (median [Q1; Q3]: 764.1 [641.8; 819.8] vs. 672.2 [581.2; 749.4] min/day, *p* = 0.046; ES = 0.262). No statistically significant differences were observed for gait speed and distance walked in the 6MWT (*p* > 0.05). A detailed description of PA and physical capacity characteristics for both groups can be found in Table 3.

**Table 3:** Physical activity levels and exercise capacity of PA App Users and Non-PA App Users.

**Variable**	**PA App Users** **(*n* = 15)**	**Non-PA App Users** **(*n* = 44)**	***P*-value**	**Effect** **size**
**Physical activity**				
BPAAT	4.0 [2.0; 6.0]	2.0 [0.0; 4.0]	0.016^*^	0.313
Accelerometry (min/week)				
Sedentary behavior	764.1 [641.8; 819.8]	672.2 [581.2; 749.4]	0.046^*^	0.262
Light PA	88.7 [65.1; 153.7]	116.9 [94.0; 212.7]	0.262	0.147
Moderate PA	31.0 [7.0; 44.1]	19.4 [8.8; 53.0]	0.372	0.117
Vigorous PA	0.5 [0.2; 2.5]	0.2 [0.1; 1.0]	0.095	0.220
MVPA	34.2 [7.7; 50.8]	19.5 [9.0; 57.2]	0.253	0.150
Step per day	5242 [2648; 8608]	4420 [3329; 9430]	0.306	0.134
**Physical capacity**				
6MWD (m)	456.2 ± 110.0	432.2 ± 82.2	0.212	0.187
Gait speed (m/s)	1.4 [1.3; 1.9]	1.5 [1.2; 1.6]	0.403	0.113

*Results are presented as mean ± standard deviation for variables that follow a normal distribution, and as median [P25; P75] for variables not following a normal distribution. Accelerometry-based data are a mean of 7 days. 6MWD, 6-min walking distance; BPAAT, Brief Physical Activity Assessment Tool; MVPA, moderate-to-vigorous physical activity; PA, physical activity. Sample size per variable is in Supplementary Material 1*.

* *p < 0.05*.

## Discussion

Our findings suggest that people with COPD who use mobile apps (besides the standard interfaces provided with the phone) are younger and have higher physical capacity compared with their peers that do not use mobile apps. Those who used PA apps reported spending more time in MVPA, although objective accelerometry data showed no significant differences in MVPA between groups and higher time spent in sedentary behaviors in PA-Apps Users.

Similar to studies on healthy people (13) and in other chronic conditions, such as tinnitus (30) and allergic respiratory diseases (31), in our study people using apps were younger. However, our sample was older than the ones in other studies (43-81 years old vs. 17-54 years old) (30, 31). This finding suggests that, although mobile apps are mainly used by youngers, an increasing rate of older people is also keen to use this technology. Indeed, 73% of our sample reported being interested in using a COPD-specific app for PA promotion if it was available to them. We did not explore reasons for not using PA apps in Non-App Users who reported being interested in using a COPD-specific app for PA. Such investigation is needed to drive the development of more attractive and relevant apps for this population and to optimize the uptake of these digital solutions.

Participants with COPD using apps presented higher physical capacity than those not using apps, as shown by walking greater distances in the 6MWT and faster in the gait speed test. This difference could be related to App users being younger, as significant correlations between age and gait speed became apparent from our data (data not showed) and have been reported in the literature (32, 33). Specifically, Zeng et al. have reported age as a significant independent predictor of the distance walked in the 6MWT in people with COPD (adjusted R^2^ = 0.445, *p* < 0.01) (32).

According to the BPAAT, PA App users reported being significantly more active than Non-PA App Users. These results were not confirmed by the time spent in MVPA assessed with the accelerometer. In fact, the accelerometer data showed that PA App Users spend significantly more time in sedentary activities than Non-PA App Users while no significant differences were observed in accelerometer-based MVPA (Table 3). These results seem to point toward an overestimation of the PA levels on the BPAAT when compared with the objective measure (accelerometry), which is a known limitation of PA questionnaires (34). Although validated for COPD and quick and easy to implement, the BPAAT was only weakly to moderately correlated with accelerometry and thus caution is needed when using it in clinical practice (22).

The increased time spent in sedentary behavior by PA App users is also an important finding for mHealth developers. Evidence indicates that, in the general population, sedentary behavior is associated with detrimental health consequences such as developing diabetes and cardiovascular conditions (35) and, in people with COPD, it has shown to be an independent predictor of mortality (35). Nevertheless, the majority of the mHealth apps for PA promotion in COPD aim only to increase total PA levels (8, 12) and little attention has been given to reducing the time these individuals spend in sedentary behaviors, which may be as relevant as increasing PA and a more feasible goal in people with COPD (36).

The success of mHealth apps in improving PA and reducing sedentary time in COPD could potentially be increased if apps were used for tele-coaching (8, 37). Previous research has shown positive results using tele-coaching for behavior change, but it has also been recognized that this type of intervention is not a “one size fits all” (9, 38). Results of the present study are useful to inform the decision-making process of features to be considered in tele-coaching interventions provided through mHealth apps, taking into consideration the demographics, clinical and PA characteristics of the target audience.

### Limitations

This study has some limitations that need to be acknowledged. This was a secondary analysis of a larger trial and thus sample size was not calculated for the specific aims of this study. The study may be underpowered to find significant differences in the outcomes of interest. Nevertheless, effect sizes, a measure that is independent of the sample size, have also been calculated and reported throughout the paper, showing moderate effects for all statistically significant differences. By reporting this detailed information, we hope that the results of this study will be used for sample size calculation by future larger trials in the field.

Most participants were males (*n* = 43; 73%) and classified as GOLD A (*n* = 28, 51%). An analysis published in The Lancet Global Health in 2018 (39) found that, across most countries, women are less active than men (global average of 31.7% for inactive women vs. 23.4% for inactive men) and it is also known that greater severity of disease and symptoms is related to lower PA levels and physical capacity (40). Thus, differences found between groups may not be generalizable to women and people in more severe levels of the GOLD ABCD classification.

## Conclusion

Our findings suggest that people with COPD who use mobile apps are younger and have higher physical capacity compared with their peers that do not use mobile apps. Those who specifically use PA apps seem to spend more time in sedentary behaviors and self-report more time in MVPA. Future studies should investigate possible explanations for these findings to inform the development and implementation of future mHealth apps.

## Data Availability Statement

The raw data supporting the conclusions of this article will be made available by the authors, without undue reservation.

## Ethics Statement

The studies involving human participants were reviewed and approved by the Ethics Committees of Polytechnic of Leiria, the Hospital Centres of Leiria and Baixo Vouga, the District Hospital of Figueira da Foz, the Northern Lisbon University Hospital Centre, and the Regional Health Administration of Central Portugal. The patients/participants provided their written informed consent to participate in this study.

## Author Contributions

JC conceived and designed the work, was responsible for obtaining the funding, and ensured project administration and resources. JC, AM, NM, CS, FS, JR, BPC, and AO obtained the funding. SF and NH performed data collection. BPC assessed participants for eligibility criteria and referred them to the study. DB and CB provided consultancy during the project development. SF, NH, AO, and JC performed data analysis and interpreted the data. SF, NH, and AO drafted the manuscript. All authors critically revised the manuscript, ensured accuracy and integrity of the work, approved the final version to be published, and agreed to be accountable for all aspects of the work.

## Funding

This work was funded by FEDER through COMPETE2020—Programa Operacional Competitividade e Internacionalização (POCI-01-0145-FEDER-028446) and by national funds (OE) through Fundação para a Ciência e Tecnologia (FCT/MCTES) (PTDC/SAU-SER/28446/2017), within the project OnTRACK—On Time to Rethink Activity Knowledge: a personalized mHealth coaching platform to tackle physical inactivity in COPD, and by Portuguese national funds provided by FCT (UIDB/05704/2020 and UIDB/04501/2020). SF was financially supported by the PhD fellowship DFA/BD/6954/2020, funded by FCT, MCTES, FSE, Por_Centro, and UE.

## Conflict of Interest

The authors declare that the research was conducted in the absence of any commercial or financial relationships that could be construed as a potential conflict of interest.

## Publisher's Note

All claims expressed in this article are solely those of the authors and do not necessarily represent those of their affiliated organizations, or those of the publisher, the editors and the reviewers. Any product that may be evaluated in this article, or claim that may be made by its manufacturer, is not guaranteed or endorsed by the publisher.
