# Supplementary material for: Phenotyping Adopters of Mobile Applications Among Patients With COPD: A Cross-Sectional Study
Source: Front Rehabil Sci. 2021 Nov 4;2:729237. doi: 10.3389/fresc.2021.729237 (PMC9397739; doi:10.3389/fresc.2021.729237)
Supplement: Supplementary file 1 [file Table_1.DOCX]

Supplementary Material

Phenotyping adopters of mobile applications among patients with COPD: a cross-sectional study

Sofia Flora, Nádia Hipólito; Dina Brooks, Alda Marques, Nuno Morais, Cândida G. Silva, Fernando Silva, José Ribeiro, Rúben Caceiro, Bruno P Carreira, Chris Burtin, Sara Pimenta, Joana Cruz, Ana Oliveira

**Supplementary material 1 – Sample size per variable.**

| Variable | n |
| --- | --- |
| 6MWD | 58 |
| 6MWD%pred | 58 |
| ABCD Assessment Tool | 55 |
| Education Level | 59 |
| Accelerometry | 58 |
| Age | 59 |
| BMI | 59 |
| Brief-PA | 59 |
| CAT | 59 |
| CCI | 58 |
| CIS20-P | 51 |
| FEV1 | 58 |
| FEV1%pred | 58 |
| Gait speed | 55 |
| GOLD FEV1 | 59 |
| Interest in trying new app | 59 |
| mMRC | 59 |
| Sex | 59 |
| Use/not use apps | 59 |
| Use/not use PA apps | 59 |

**Legend:** 6MWD, Six-Minute Walking Distance; BMI, Body Mass Index; CAT, COPD Assessment Test; CCI, Charlson Comorbidity Index; CIS20, Checklist of Individual Strength; FEV_1_, Forced Expiratory Volume in First Second; GOLD, Global Initiative for Obstructive Lung Disease; mMRC, Modified Medical Research Council. Variables are presented in alphabetical order.

**Supplementary material 2 - Sociodemographic and clinical characteristics of PA App Users and Non-PA App Users.**

| **Characteristics** | **PA App Users (n=15)** | **Non-PA App Users (n=44)** | **p-value** | **Effect**  **size** |  |
| --- | --- | --- | --- | --- | --- |
| Age (years) | 64.1 ± 10.9 | 67.0 ± 7.3 | 0.067 | 0.785 |  |
| FEV_1_% pred | 51.7 ± 24.6 | 47.6 ± 17.4 | 0.469 | 0.219 |  |
| BMI, mean ± SD | 26.8 ± 4.5 | 25.6 ± 4.9 | 0.480 | 0.255 |  |
| Sex, n (%)  Female  Male | 3 (20)  12 (80) | 13 (30)  31 (70) | 0.771 | 0.094 |  |
| Education Level, n (%)  Primary  Secondary  Undergraduate  Graduate | 9 (59.9)  2 (13.3)  1 (6.7)  3 (20) | 30 (68.2)  9 (20.5)  1 (2.3)  4 (9.1) | 0.117 | 0.863 |  |
| GOLD Classification, n (%)  GOLD 1  GOLD 2  GOLD 3  GOLD 4 | 1 (6.7)  6 (40)  6 (40)  2 (13.3) | 1 (2.3)  18 (41.9)  18 (41.9)  6 (14) | 0.890 | 0.180 |  |
| GOLD ABCD Tool, n (%)  GOLD A  GOLD B  GOLD C  GOLD D | 6 (46.2)  3 (23.1)  2 (15.4)  2 (15.4) | 22 (52.4)  5 (11.9)  6 (14.3)  9 (21.4) | 0.773 | 0.247 |  |
| CCI, n (%)  Mild  Moderate  Severe | 3 (20.0)  7 (46.7)  5 (33.3) | 7 (16.3)  28 (65.1)  8 (18.6) | 0.408 | 0.249 |  |
| CAT Total Score | 13.46 ± 7.7 | 11.8 ± 8.4 | 0.232 | 0.127 |  |
| CIS20-P Total | 59.6 ± 21.7 | 62.4 ± 24.2 | 0.107 | 0.176 |  |
| mMRC (median [Q1; Q3]) | 1 [1; 2] | 1 [1; 2] | 0.240 | 0.305 |  |

**Legend:** Results are presented as mean±standard deviation, unless otherwise stated.

BMI, body mass index; CCI, Charlson Comorbidity Index; CAT, COPD Assessment Test; CIS20P, Checklist of Individual Strength FEV_1_, forced expiratory volume in the first second; GOLD, Global Initiative for Obstructive Lung Disease; mMRC, Modified Medical Research Council.
